# Decreased Incidence of Intraventricular Hemorrhage in Extremely Low Birth Weight Infants Using Customized Circulatory Management to Evaluate the Hemodynamic Change of Patent Ductus Arteriosus

**DOI:** 10.3389/fped.2021.711871

**Published:** 2021-09-30

**Authors:** Wan-Heng Huang, De-Ming Li, Chung-Ting Hsu, Yi-Hsuan Lin, Ya-Chi Hsu, Teh-Ming Wang, Ming-Chih Lin

**Affiliations:** ^1^Children's Medical Center, Taichung Veterans General Hospital, Taichung, Taiwan; ^2^Division of Pediatric Neonatology, Da Chien General Hospital, Miaoli City, Taiwan; ^3^School of Medicine, National Yang-Ming University, Taipei, Taiwan; ^4^Department of Food and Nutrition, Providence University, Taichung, Taiwan; ^5^School of Medicine, Chung Shan Medical University, Taichung, Taiwan; ^6^School of Medicine, National Chung Hsing University, Taichung, Taiwan

**Keywords:** extremely low birth weight infants (ELBW infants), patent ductus arteriosus (PDA), Intraventricular hemorrhage (IVH), customized circulatory management, dobutamine

## Abstract

**Background:** In extremely low birth weight (ELBW) infants, the patent ductus arteriosus (PDA) with left-to-right shunt and an increase in systemic artery resistance may cause increasing preload and afterload of the left ventricle. The immature myocardium in ELBW infants has a limited ability to respond to the change, which leads to hemorrhagic complications. In this study, we detected the hemodynamic change of cardiac performance and applied a clinical strategy to prevent PDA-associated hemorrhagic complications in ELBW infants.

**Methods:** We enrolled ELBW infants at a single medical center in Taiwan. The customized circulatory management was performed by echocardiography after birth until the PDA closed. Inotropic agents were administrated according to the requirements of hemodynamic parameters or clinical conditions. The primary outcomes were hemorrhagic complications including pulmonary hemorrhage and intraventricular hemorrhage (IVH) greater than grade II. The secondary outcomes were the rate of surgical ligation of PDA, mortality, necrotizing enterocolitis, and bronchopulmonary dysplasia.

**Results:** A total of 20 ELBW infants were evaluated by customized circulatory management from 2019 to 2020. We reviewed 35 ELBW infants born between 2017 and 2018 in our hospital, who served as the non-management group. The management group had a significantly lower incidence rate of IVH greater than grade 2 (*p* = 0.02). Other outcomes showed no significant differences. Dobutamine was prescribed in 8 cases in the management group, and end-systolic wall stress (ESWS) was significantly decreased after Dobutamine administration (*p* = 0.017).

**Conclusion:** The incidence rate of IVH greater than grade II in ELBW infants decreased after use of customized circulatory management in our study. The strategy of customized circulatory management might be an effective “early target therapy” for hemodynamically significant PDA in high-risk ELBW infants. Inotropic therapy with Dobutamine could be a useful medical choice for improving cardiac function to prevent hemorrhagic complications.

## Introduction

In pre-term infants, lower gestational age is related to lower incidence of spontaneous closure of the patent ductus arteriosus (PDA). It is more common in extremely low birth weight (ELBW) infants who have a gestational age of <28 weeks or a birth body weight <1,000 grams. Spontaneous closure of PDA occurs in less than one third of ELBW infants at day 7 after birth (1, 2). The pulmonary vascular resistance declines in the first few days of life, and then the blood flow of PDA shifts to left-to-right shunt. Increased pulmonary blood flow causes pulmonary edema and pulmonary congestion, eventually resulting in respiratory failure (3, 4). The hemodynamic change with increasing pulmonary blood flow and decreasing systemic blood flow also induces numerous clinical symptoms (5–8). The major complications of hemodynamically significant PDA has been reported, such as intraventricular hemorrhage (IVH), pulmonary hemorrhage, necrotizing enterocolitis (NEC), bronchopulmonary dysplasia (BPD), and mortality (3, 4, 6, 8–14).

A number of studies have investigated the hemodynamic change between fetus and newborn. According to previous research, the placenta has the lowest vascular resistance in the fetal stage. The resistance in the systemic artery increases suddenly after birth because of an interruption from the placenta. It causes an increase in the afterload of the left ventricle (15–17). An increase in the pre-load of left ventricle was also observed due to the left-to-right shunt in blood flow of PDA during the decline of pulmonary vascular resistance (7, 18). The immature myocardium, especially in ELBW infants, has a limited response to the increase in preload and afterload. It could induce hemorrhagic complications, including IVH and pulmonary hemorrhage (3, 6). Many echocardiographic parameters have been used to evaluate the hemodynamic change in pre-term infants. However, the stress-velocity relationship is a relatively independent index for evaluation of cardiac function (3, 6, 17, 19–21). Toyoshima et al. used customized circulatory management to evaluate cardiac function based on the stress-velocity relationship aimed at decreasing the mortality rate and incidence of severe IVH in ELBW infants (3). In our study, we used a similar circulatory management and clinical strategy to evaluate the hemodynamic change of PDA, and tried to improve our PDA-associated outcomes in ELBW infants.

## Materials and Methods

### Study Design and Patients

This was a prospective cohort study at a single medical center in Taiwan. We enrolled extremely low birth body weight (ELBW) infants with birth weight of <1,000 g born at the neonatal intensive care unit (NICU) of Taichung Veterans General Hospital between 1 January, 2019 and 31 December, 2020. Babies with chromosomal abnormalities, complex congenital heart diseases, or multiple anomalies were excluded. All infants' demographic data including gender, gestational age, birth body weight, Apgar scores at the 1st and 5th min, percentage of body weight loss after birth, Ibuprofen usage, Surfactant usage, inotropes usage, intubation, days of intubation, post-menstrual age (PMA), and body weight at discharge were collected. This study design and protocol were approved by the Institutional Review Board of Taichung Veterans General Hospital. All participants' parents had to sign the informed consent before the study.

### Echocardiographic Managements

We collected the cardiac parameters by echocardiography and clinical data every 12 h after birth until PDA closed. Because the closure of PDA of ELBW infants was variable clinically, we set the endpoint of our study as PDA closure was documented by echocardiography in two consecutive measurements. The measured cardiac parameters in customized circulatory management included left ventricular internal dimension-diastole (LVIDd, cm), left ventricular internal dimension-systole (LVIDs, cm), posterior wall thickness at end-systole (Hes, cm), left ventricular ejection time (ET, sec), RR interval (RR, sec), and end-systolic pressure (Pes, mmHg). The left ventricle rate-corrected mean velocity of circumferential fiber shortening (mVcfc, circ/s) and end-systolic wall stress (ESWS, g/cm^2^) were calculated using the formulae for these multiple cardiac parameters (3, 22, 23). The mVcfc was an index of left ventricular pumping function. The ESWS was an index of left ventricular afterload or systemic vascular resistance (3, 22, 23). We followed a series of values of mVcfc and ESWS in every infant enrolled in the trial.

### Strategy

In our NICU, general measures provided to the ELBW infants included 90% humidified incubator in the first week, initial daily fluid with 80 ml/kg per day, adjusting amount of intravenous fluid according to body weight every 8 h, and minimal ventilatory support ensuring adequate oxygenation (pulse oximetry saturation between 90 and 95 percent) and allowing permissive hypercapnia (partial pressure of carbon dioxide between 40 and 60 mmHg in arterial blood). According to previous research, appropriate cardiac function in ELBW infants was described as the value of mVcfc above 0.8 circ/s and ESWS was below 40 g/cm^2^ (3). Inotropic therapy using Dobutamine with an initial dose of 2 μg/kg/min was administrated if mVcfc < 0.8 circ/s, ESWS > 40 g/cm^2^, or an upward trend of ESWS was observed during measurement. Inotropic therapy using Dopamine was administrated when mean artery pressure was lower than normal for the gestational age.

The hemodynamically significant PDA was defined according to clinical manifestations or echocardiographic parameters in our NICU. The clinical findings included systolic heart murmur, hyperactive precordial pulsation, bounding pulses, persistent tachycardia, persistent tachypnea, cardiomegaly, or hepatomegaly. The echocardiographic findings included left atrial or left ventricular dilatation, reversed diastolic flow in descending aorta, mVcfc < 0.8 circ/s, or ESWS > 40 g/cm^2^. For those infants who had a hemodynamically significant PDA, pharmacologic intervention with a course of Ibuprofen was given. If the patients failed to respond to an initial course of therapy, administration of the second course of Ibuprofen was considered clinically. The surgical ligation of PDA was prescribed as rescue management if the patients failed to respond to two courses of medical treatment or suffered one of the serious complications, including progressive anuria, progressive hypotension, NEC, severe IVH, or pulmonary hemorrhage.

### Outcomes

The primary outcomes were the rates of hemorrhage complications including pulmonary hemorrhage and IVH greater than grade II. The additional outcomes were the rates of surgical ligation of PDA, BPD, definite NEC, and mortality. The BPD was defined as a single entity of oxygen requirement either at 28 postnatal days or 36 weeks postmenstrual age (24). Definite NEC was defined as NEC with modified Bell's stage ≥ IIA clinically (25).

### Statistical Analysis

Continuous data were expressed as mean and standard deviation, and categorical data were expressed as frequency and percentage. Continuous data were compared using the Mann-Whitney *U*-test, and categorical data were compared using Pearson's chi-square test. Fisher's exact test was applied if any cell of categorical data was <5. The Wilcoxon rank-sum test was used to compare the values of mVcfc and ESWS before and after Dobutamine administration. Statistical significance was defined as a *p* < 0.05. Analyses were performed using statistical software IBM SPSS Statistics for Windows, version 25.0. (Armonk, NY: IBM Corp.).

## Results

**Figure 1:**
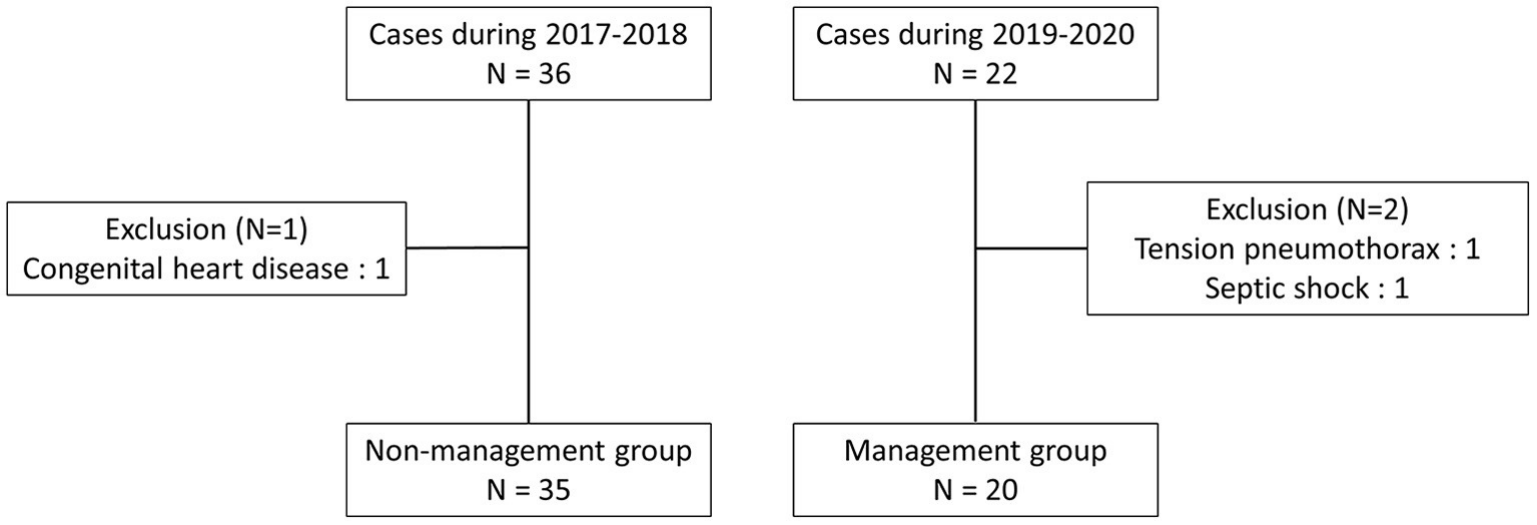
The study flowchart of enrolled ELBW infants.

A total of 22 ELBW infants were admitted to the NICU of Taichung Veterans General Hospital from January 1, 2019 to December 31, 2020. We excluded 2 cases because both of them expired within 48 h after birth. One suffered from septic shock because of chorioamnionitis, and the other suffered tension pneumothorax. The causes of death were not associated with PDA in both cases. A total of 20 cases that received customized circulatory management during the study period were enrolled. We also retrospectively reviewed ELBW infants who were admitted to the NICU of Taichung Veterans General Hospital from January 1, 2017 to December 31, 2018 using the same enrolment criteria and these infants served as the non-management group (the control group). One case was excluded due to congenital heart disease. There were 35 cases in the non-management group, which were included in the final analysis. The study flowchart is shown in Figure 1. The demographic data are shown in Table 1. Dobutamine was administrated in 8 of 20 cases in the management group, and none of the cases in the non-management group. There were no significant differences in gender, Ibuprofen usage, Surfactant usage, Dopamine usage, intubation, days of intubation, gestational age, birth body weight, Apgar scores at the 1st and 5th min, percentage of body weight loss after birth, postmenstrual age, and body weight at discharge between the two groups.

**Table 1:** Demographic data in the non-management and management groups.

	**Non-management**	**Management**	***P*-value**
	**(2017–2018)**	**(2019–2020)**	
	***N* = 35**	***N* = 20**	
Male	21(60.0%)	10 (50.0%)	0.47
Ibuprofen usage	22 (62.9%)	14 (70.0%)	0.59
Surfactant usage	22(62.9%)	9 (45.0%)	0.2
**Inotropes**			
Dopamine	16 (45.7%)	7 (35.0%)	0.44
Dobutamine	0 (0.0%)	8 (40.0%)	0
Intubation	33 (94.3%)	16 (80.0%)	0.1
Days of intubation	19.2 ± 20.8	24.7 ± 38.1	0.77
Gestational age (weeks)	25.7 ± 1.9	26.6 ± 1.6	0.09
Birth body weight (grams)	770.1 ± 156.5	815.0 ± 133.8	0.23
Apgar score at 1st min	3.3 ± 1.7	3.8 ± 1.3	0.21
Apgar score at 5th min	5.8 ± 1.7	6.7 ± 1.5	0.1
Percentage of body weight loss (%)	11.1 ± 5.7	11.4 ± 5.3	0.89
Discharge PMA (weeks)	40.1 ± 11.0	44.3 ± 4.6	0.05
Discharge body weight (grams)	2,970.9 ± 1,799.5	3,376.8 ± 1,083.4	0.33

*PMA, post-menstrual age*.

Pulmonary hemorrhage occurred in 4 of 35 cases (11.4%) in the non-management group and 2 of 20 cases (10.0%) in the management group (*p* = 1.00). Intraventricular hemorrhage greater than grade II was noted in 12 of 35 cases (34.3%) in the non-management group and in 1 of 20 cases (5.0%) in the management group. The rate of IVH higher than grade II decreased significantly in the management group (*p* = 0.02). The additional outcomes were not significantly different between the two groups in rates of mortality (*p* = 0.18), surgical ligation of PDA (*p* = 0.52), NEC greater than stage 2 (*p* = 1.00), and BPD (*p* = 0.24; Table 2).

**Table 2:** Outcomes in the non-management and management groups.

	**Non-management**	**Management**	***P*-value**
	***N* = 35**	***N* = 20**	
Pulmonary hemorrhage	4 (11.4%)	2 (10.0%)	1
IVH ≥ grade 2	12 (34.3%)	1 (5.0%)	0.02
Mortality	10 (28.5%)	2 (10.0%)	0.18
Surgical PDA ligation	11 (31.4%)	8 (40.0%)	0.52
NEC ≥ stage 2	14 (40.0%)	8 (40.0%)	1
Bronchopulmonary dysplasia	13 (37.1%)	4 (20.0%)	0.24

*IVH, intraventricular hemorrhage; NEC, necrotizing enterocolitis; PDA, patent ductus arteriosus*.

**Figure 2:**
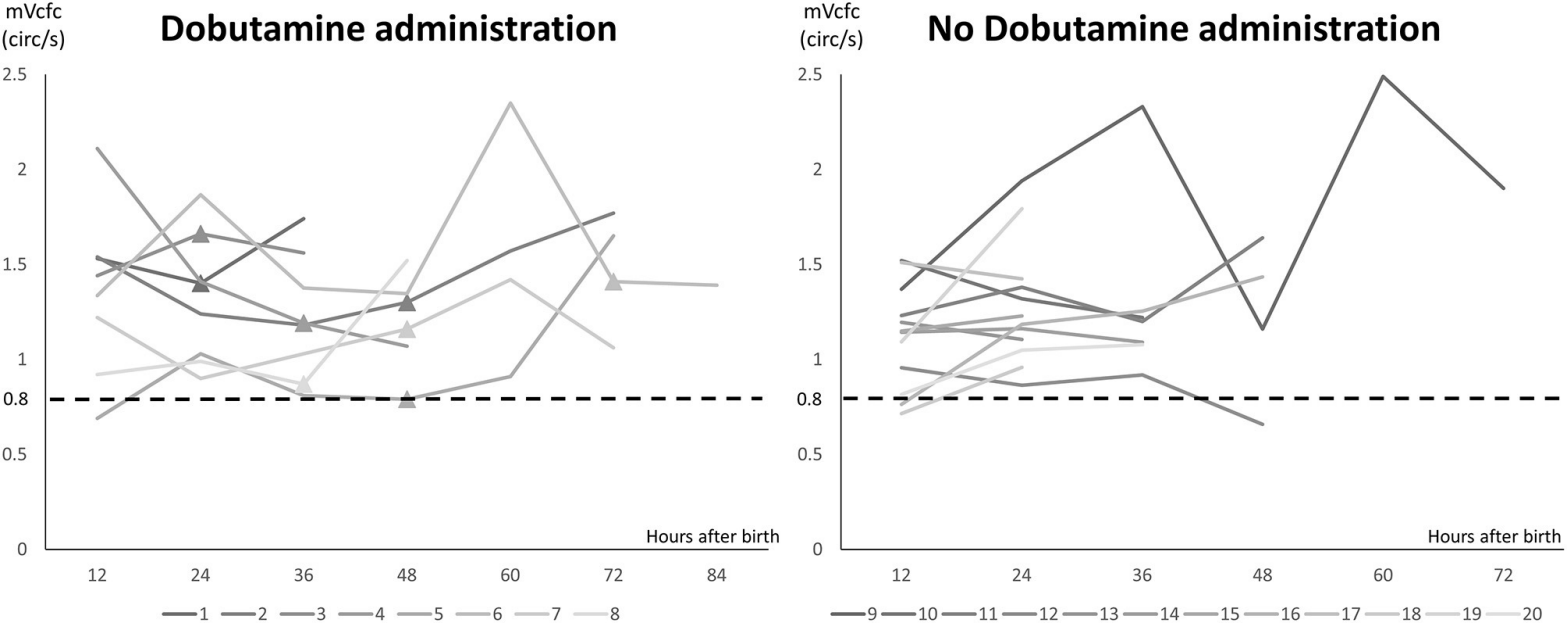
Longitudinal values of mVcfc in the management group. Twenty cases were divided into two subgroups: 8 cases with Dobutamine administration and 12 cases without Dobutamine administration. X-axis showed hours after birth, and Y-axis showed the value of mVcfc. ▴ stood for the timing of initiating Dobutamine administration.

**Figure 3:**
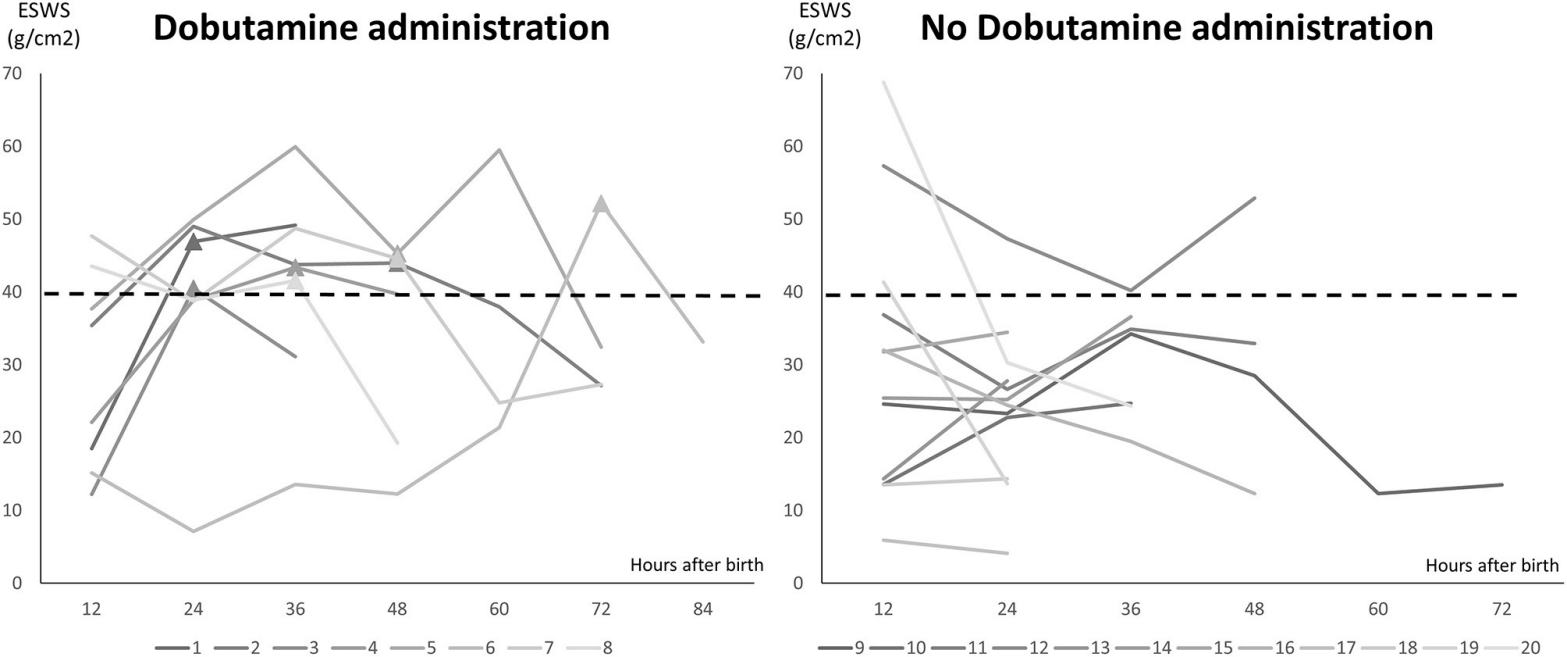
Longitudinal values of ESWS in the management group. Twenty cases were divided into two subgroups: 8 cases with Dobutamine administration and 12 cases without Dobutamine administration. X-axis showed hours after birth, and Y-axis showed the value of ESWS. ▴ stood for the timing of initiating Dobutamine administration.

**Figure 4:**
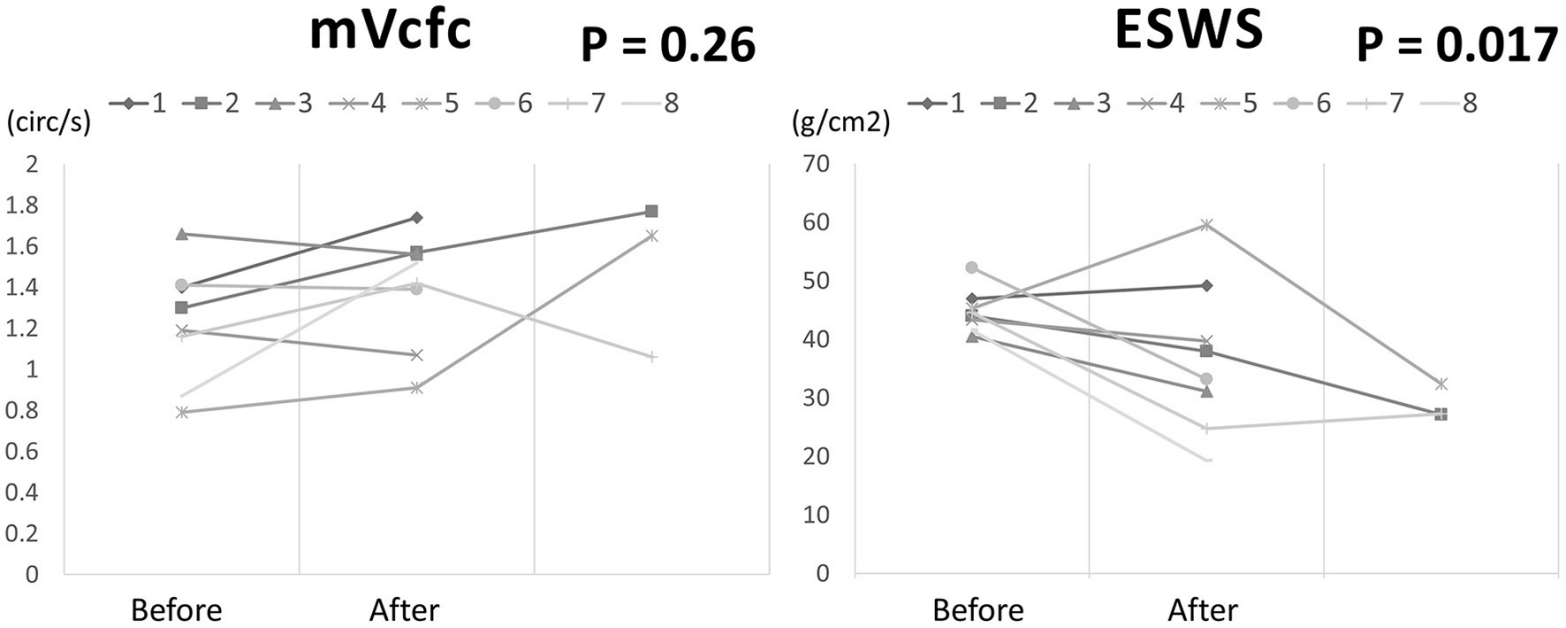
The changing of mVcfc and ESWS before and after Dobutamine administration in 8 cases of the management group.

There were 8 cases (8/20) using Dobutamine administration in the management group due to mVcfc < 0.8 circ/s, ESWS > 40 g/cm^2^, or an upward trend of ESWS during the study period. After initiating Dobutamine, the dosage would be adjusted according to the value of ESWS in the next measurement. If the value of ESWS increased, the dosage would be titrated up by 2 μg/kg/min. If the value of ESWS decreased or remained constant, we would keep the current dosage until PDA closed. Figure 2 showed all longitudinal values of mVcfc in the management group. Almost all values of mVcfc at any time were above 0.8 circ/s whether Dobutamine was administrated or not. Figure 3 showed all longitudinal values of ESWS in the management group. In 8 cases with Dobutamine administration, ESWS > 40 g/cm^2^ or an upward trend of ESWS was observed before using Dobutamine, and ESWS showed a decreasing trend in almost all cases after using Dobutamine. On the contrary, ESWS < 40 g/cm^2^ or a downward trend of ESWS was observed as time went on in 12 cases without Dobutamine administration. The value of mVcfc and ESWS were recorded before and after Dobutamine administration in each case. We used the Wilcoxon rank sum test for further analysis. There was no significant difference in value of mVcfc before and after Dobutamine usage (*p* = 0.26). However, value of ESWS decreased significantly before and after Dobutamine usage (*p* = 0.017; Figure 4).

## Discussion

In our study, we found that the incidence of IVH greater than grade II in ELBW infants declined after implementing customized circulatory management in our NICU. The other outcomes including pulmonary hemorrhage, surgical ligation of PDA, definite NEC, BPD, and mortality showed no difference between the management group and the non-management group. Dobutamine administration might be able to reduce the afterload of the left ventricle, but there was no improvement in the pumping function of the left ventricle in our study. Toyoshima et al. (3) reported introduction of customized circulatory management in ELBW infants could reduce IVH both in incidence and in severity. They found Dobutamine administration might deteriorate cardiac pumping function and even increase systemic vascular resistance in half of their patients. On the contrary, they used intravenous Nitroglycerin to reduce ESWS and increase mVcfc effectively in most patients. The intravenous form of nitroglycerin was not available in our hospital. However, ESWS reduced significantly after Dobutamine administration in our clinical practice. In our opinion, Dobutamine might be another choice for decreasing systemic vascular resistance in ELBW infants.

The recent trend of PDA management in ELBW infants is “early target treatment,” which aims to identify a hemodynamically significant PDA in specific high-risk populations as early as possible (26–30). Our study proved customized circulatory management might be an effective “early target treatment.” In clinical practice, we identified high-risk ELBW infants who had lower mVcfc or higher ESWS, and prescribed inotropic therapy with Dobutamine administration. Using a series of cardiac parameters (mVcfc and ESWS) measured by echocardiography after birth, we were able to determine an adequate timing and indication to intervene with medical treatment for PDA in ELBW infants.

The mechanism of the association between IVH and increased afterload of the left ventricle in ELBW infants was proposed in previous studies (3, 6). To deal with an increased afterload, the preload of the left ventricle is increased due to preload reserve (15, 31). Increased preload of the left ventricle may result in increased preload of the right ventricle because of tight ventricle interaction. An increase in preload of right ventricle leads to elevated central venous pressure and elevated cerebral venous pressure. As a consequence, the fragile vein around the germinal matrix is not able to tolerate the increased cerebral venous pressure, which causes IVH (3, 31–35). In addition, the left ventricle of the pre-term infant has low distensibility (15, 31), which results in increased pulmonary venous pressure and pulmonary congestion during elevation of preload of the left ventricle, ultimately leading to pulmonary hemorrhage (36–38).

A previous study reported grade II IVH in pre-term infants still leads to post-hemorrhagic hydrocephalus (39). The low-grade IVH may also affect brain tissue structure and long-term neurodevelopmental outcomes (40–42). It is for this reason we recognized IVH greater than grade II as one of primary outcomes in our study.

There were certain limitations in the study. In general, echocardiography is a highly operator-dependent skill. A previous study showed the inter-observer repeatability of echocardiographic parameters was poor (43). Operator error may have affected the accuracy of the collected data. Second, this was a single center study. Customized circulatory management was implemented for ELBW infants in our NICU during a 2-year period, which resulted in a small sample size. The effects of this treatment modality on long-term neurodevelopment and growth trends could therefore not be established in our study. Further studies with a larger sample size and a longer follow-up period are necessary.

In conclusion, using customized circulatory management might decrease the incidence of intraventricular hemorrhage in extremely low birth weight infants. Early inotropic therapy with Dobutamine administration might be helpful to reduce the afterload of the left ventricle in extremely low birth weight infants. This strategy of customized circulatory management might be an effective “early target therapy” to avoid serious hemorrhage complications of PDA in the first few days after birth. Because of the small sample size in our study, further research with larger participation is needed in the future.

## Data Availability Statement

The original contributions presented in the study are included in the article/Supplementary Material, further inquiries can be directed to the corresponding authors.

## Author Contributions

M-CL: conceptualization. W-HH and D-ML: data curation. W-HH: formal analysis and writing—original draft. C-TH: methodology and writing—review and editing. All authors contributed to the article and approved the submitted version.

## Funding

The study was supported by Taichung Veterans General Hospital research fund (TCVGH-110DHA0500728).

## Conflict of Interest

The authors declare that the research was conducted in the absence of any commercial or financial relationships that could be construed as a potential conflict of interest.

## Publisher's Note

All claims expressed in this article are solely those of the authors and do not necessarily represent those of their affiliated organizations, or those of the publisher, the editors and the reviewers. Any product that may be evaluated in this article, or claim that may be made by its manufacturer, is not guaranteed or endorsed by the publisher.
